# Investigation of the relationship between adoption of minimalist and sustainable lifestyle, and individuals’ views on artificial meat

**DOI:** 10.3389/fnut.2025.1678809

**Published:** 2025-11-25

**Authors:** Güzin Yasemin Tunçay, Nesrin Çobanoğlu

**Affiliations:** 1 Çankırı Karatekin University Faculty of Health Sciences, Çankırı, Türkiye; 2 Department of Medical History and Ethics, Faculty of Medicine, Gazi University, Ankara, Türkiye

**Keywords:** artificial meat, bioethics, environmental sustainability, minimalist lifestyle, simple living, sustainable nutrition

## Abstract

**Background:**

It is thought that due to fact that the world population is increasing and that the prediction that the world’s resources will be inadequate, the need for sustainable lifestyles, sustainable consumption, sustainable nutrition and therefore alternative food solutions such as artificial meat as a sustainable protein source will increase. In sustainable nutrition, individuals consume only what they need; in other words, they have a healthy and balanced diet. Artificial meat is also considered a form of nutrition that is environmentally sustainable, because its negative environmental effects are minimal, and it ensures food safety. The Minimalist Lifestyle, where simple living and consumption only for needs are at the forefront, is also closely related to environmental sustainability goals. A minimalist lifestyle can be considered as a form of sustainable lifestyle. Therefore, it is thought that sustainable nutrition, the minimalist lifestyle, and the consumption of artificial meat have the potential to support environmental sustainability, and that there is a significant relationship between these three themes.

**Methods:**

In this study, the possible relationship between individuals adopting a minimalist and sustainable lifestyle, and their views on artificial meat were investigated. Two scales were used in the study. One of them is the Scale for Determining the Minimalist Lifestyle from the Point of Environmental Bioethics, and the other one is the Scale for Behaviors towards Sustainable Nutrition. In addition to these scales, the participants were asked open-ended questions about artificial meat. It was also investigated whether individuals’ minimalist tendencies positively affected their perspectives on artificial meat with survey and analysis methods.

**Results:**

In this study, it was determined that the majority of people who embrace minimalist lifestyle also embrace a sustainable diet. It was investigated that 65% of the participants did not have knowledge about artificial meat and 83.4% did not have knowledge about the production process. Regarding consumption preferences, 87.6% indicated that they would not choose to consume artificial meat, and approximately half of the participants (51.6%) either did not or would not hold a positive opinion about it. Furthermore, it was found that the preference for artificial meat consumption is lower among participants embracing a minimalist lifestyle. Participants have doubts about the health effects of artificial meat consumption. These results may be influenced by the cultural and religious beliefs of Turkish society.

**Conclusion:**

The research results indicate that some cultures may have a negative view of artificial meat. It is believed that awareness of the potential beneficial effects of artificial meat on the ecosystem could gradually change this perspective over time. Due to the growing world population and decreasing global resources, it is expected the need for alternative and sustainable protein sources will increase in future years. In this context, the issue of artificial meat is expected to become increasingly prominent. Various bioethical issues and dilemmas are predicted to arise. Therefore, further research should be conducted on this topic.

## 1 Introduction

It is predicted that world resources will not be enough for the increasing world population in the future. The reason for artificial meat production is the same as GMO (genetically modified organisms) aimed at feeding the increasing world population (1–3). As mentioned in the literature, there is more than one method to produce artificial meat. In one of the methods, cells are multiplied in a laboratory environment. In the second method, the animal’s genomes are changed in the laboratory, and meat is obtained from this genetically modified meat (4–7). In the third method, plant-based meat is produced using soy, wheat, peas, and beans. These vegetarian/vegan meats are currently sold in many countries. In the fourth method, meat is obtained by cloning the animal. Since such meat is also man made, it is considered as artificial meat (4). The review of the literature revealed that the meat produced in the first two methods is considered as artificial meat and evaluations are made based on them. However, regardless of the method it is produced, artificial meat is accepted as an environmentally friendly, sustainable and suitable alternative product that will be enough to feed the increasing population, because in all these methods, the use of animals decreases, which reduces the negative effects of animal husbandry on nature. In addition, it is stated that more research should be conducted on this issue and that it will take at least 10 years before artificial meat is offered for consumption (4–8).

Especially when the first two production methods are considered, there are differences between artificial meat and traditional meat. First, it is thought that artificial meat production will be less costly. However, artificial meat will have a paler color than traditional meat has. Since this might have a negative effect on the consumer, natural dyes such as sugar beet, or hemoglobin separated from the animal’s blood, are used in the production process as a solution. In addition, there is less animal slaughter in artificial meat production, a smaller carbon footprint, less water consumption, less risk of pesticide exposure, and less waste generation, which gains artificial meat the environmental sustainability feature (3, 8). While the issue of animal rights and welfare is discussed in traditional meat production, there will be considerations and concerns about legislation and social perception regarding the production of artificial meat (8). It is thought that with the production of artificial meat, animal rights will be protected, the damage to the ecosystem will be reduced, and therefore an environmentally sustainable protein source will be provided (3, 5, 6, 9). Therefore, it is possible to evaluate artificial meat consumption within the scope of sustainable nutrition. Sustainable nutrition is defined as “a diet that protects the ecosystem, biodiversity, and food security and quality of life of future generations (10).” Animal husbandry has negative effects on the ecosystem due to the consumption of meat and secondary animal products. In several studies, it has been indicated that not consuming meat or consuming it less is healthy (11–15). It is proposed that sustainable healthy nutrition should be adopted in order to end animal exploitation, to combat environmental problems caused by animal husbandry and to be healthy (16, 17).

Minimalism can also be defined as a philosophy of life, a lifestyle, through which what is important for a person is determined, and things that are not important and only create excess and clutter can be avoided. In a Minimalist Lifestyle, consumption only for needs comes to the fore, and instead of shopping things used for short term for pleasure, which creates addiction and leads to the wasting of time and money, shopping for things with long-term benefits that will bring happiness is recommended (18–20). In a Minimalist Lifestyle, a person only has as few items as necessary. For example, they do not renew their mobile phone unless it breaks, and the individual does not buy an item that they will not use (21). Expressions such as “simple life,” “simple living” and “zen style living” are also used to define minimalist lifestyle (6, 19, 20, 22, 23). This lifestyle, which is less consuming, provides not only real happiness but also an environmentally friendly, sustainable life (19, 21). Sustainable lifestyle has many aspects with nutrition and consumption patterns. One of the key dimensions of a sustainable lifestyle is nutrition and consumption habits. In this context, the concepts of sustainable nutrition and sustainable consumption come to the forefront. Since the minimalist lifestyle is based on consuming only what is needed, it overlaps with the concept of sustainable consumption. Sustainable eating refers to eating only what is necessary and in a balanced way. This approach prevents the consumption of global resources extravagantly and contributes to the healthy lives of today’s generation, and ensures the right of future generations to meet their needs. Artificial meat is also considered a sustainable protein source, a product of sustainable nutrition. Thus, it is possible to assume that minimalist lifestyles, sustainable nutrition, and artificial meat consumption are interconnected. Although artificial meat production is on the agenda, its production has not yet become widespread, so the number of people consuming this product is very few. However, given the fact that artificial meat production will be inevitable in the future, it is obvious that opinions of consumers will be of great importance. Although there are four different production methods of artificial meat, when artificial meat is mentioned, two of these production methods generally come to mind, but during the data collection process in the present study, the participants were not informed about the production methods of artificial meat, and only the concept of “artificial meat” was used.

In the present study, it was aimed to obtain the participants’ opinions on artificial meat according to their minimalist lifestyle and sustainable nutrition behaviors, and to discover the perspectives of individuals who adopted a sustainable lifestyle on artificial meat consumption for the same reasons.

## 2 Materials and methods

### 2.1 Population and sample of the study

Individuals who were over the age of 18 and were economically independent comprised the population of the study. The reason why individuals who were economically independent were chosen was that they were asked questions about their lifestyle and that they could independently determine their own lifestyle. The study sample was selected using the snowball (chain) sampling method, a type of purposive sampling method. Of the individuals who were reached, those who agreed to participate in the study were included in the study sample. In this calculated the sample size using Cohen’s effect size of 0.3, which is considered a medium effect size. Since no similar prior studies were available, a medium effect size was chosen as a reasonable estimate. Based on the power analysis conducted, the target was to reach a minimum sample size of 254 participants. A total of 321 individuals were contacted, of whom 283 met the inclusion criteria and provided valid responses. Due to the cultural and religious significance attributed to meat consumption in Turkish society, examining the level of acceptance of innovative food technologies such as artificial meat within this context is considered important for revealing how these perspectives influence consumer behavior.

The distribution of the participants according to their demographic characteristics was given in Table 1. Of them, 21.2% were in the age group of ≤35 years, 25.4% were between the ages of 36 and 45 years, 23% were between the ages of 46 and 55 years, 30.4% in the age group of ≥56 years, 71.7% were women, 72.1% were married, 70.7% had children, 49.5% were university graduates, 19.1% were academicians, 11% were physicians, 10.2% were teachers, 17% were self-employed, 41.3% perceived their income level as good and 53.7% perceived their income level as moderate (Table 1).

**Table 1:** Distribution of demographic characteristics of the participants.

Characteristics		*n*	%
Age range (years)	≤35 36–45 46–55 ≥56	60 72 65 86	21.2 25.4 23.0 30.4
Sex	Women Men	203 80	71.7 28.3
Marital status	Married Single	204 79	72.1 27.9
Having children	Yes No	200 83	70.7 29.3
The number of children*	1 2 3	88 94 17	44.2 47.2 8.5
Educational status**	Senior high school University Master’s degree	14 140 129	4.9 49.5 45.6
Profession	Academician Self-employed Physician Teacher Retired Engineer Civil Servant Nurse Other	54 48 31 29 28 27 18 16 32	19.1 17.0 11.0 10.2 9.9 9.5 6.4 5.7 11.3
Perceived income status	Good Moderate Not good	117 152 14	41.3 53.7 4.9
Total		283	100

*One participant did not answer the question. **One participant with primary school education and one participant with junior high school education were added to the senior high school education section.

### 2.2 Data collection tools

The participants administered a data collection form consisting of four sections. The first section is on their demographic characteristics. In the second section, they are asked questions about their opinions on artificial meat (e.g., Do you have any information about artificial meat? Could you prefer to consume artificial meat? Do you think artificial meat production would be beneficial or harmful? Please write down your opinions). The third section includes the Scale for Determining the Minimalist Lifestyle from the Point of Environmental Bioethics. The last section includes the Scale for Behaviors toward Sustainable Nutrition. The Scale for Determining the Minimalist Lifestyle from the Point of Environmental Bioethics (SDMLPEB) was developed by Tunçay and Çobanoğlu (24). The scale SDMLPEB can be administered to adult individuals with economic independence. The SDMLPEB which is used to measure the Minimalist Lifestyle of an individual in terms of environmental bioethics consists of 25 items, 14 of which are reversed scored. Responses given to the items are rated on a five-point likert type scale ranging from 1 to 5 (“Never,” “Rarely,” “Sometimes,” “Often” and “Always”). The SDMLPEB consists of the following three dimensions: “Minimalist Lifestyle,” “Sustainable Environment” and “Time Management.” Each dimension is related to environmental sustainability both directly and indirectly. The Minimalist Lifestyle dimension includes questions on purchasing unnecessary items and consuming things not according to the need but according to the desire. The Sustainable Environment dimension includes items directly related to the subject. The Time Management dimension includes items on using digital platforms. In this respect, it is possible to evaluate the Time Management dimension within the scope of digital waste. The lowest and highest possible scores that can be obtained from the scale “SDMLPEB” are 25 and 125, respectively. The higher the score obtained from the scale (SDMLPEB) is, the more minimalist the lifestyle the respondent adopts is.

The Scale for Behaviors toward Sustainable Nutrition (SBTSN) was developed by Garipoglu et al. (25). Since the authors of the Scale for Behaviors toward Sustainable Nutrition allow its use without getting permission provided that their names are cited and that it is used only for scientific research, permission was not obtained (26). The SBTSN consists of 29 items and the following 4 dimensions: Food Preference (Items 1, 2, 3, 4, 5, 6), Food Waste Reduction (Items 7, 8, 9, 10, 11, 12, 13, 14, 15), Seasonal and Local Nutrition (items 16, 17, 18, 19, 20, 21, 22, 23) and Food Purchase (items 24, 25, 26, 27, 28, 29). Responses given to the items are rated in a five-point Likert-type scale ranging from 1 to 5 (“1: never,” “2: rarely,” “3: sometimes,” “4: often” and “5: always”). All the items are positively keyed items. The lowest and highest possible scores that can be obtained from the SBTSN are 29 and 145, respectively. Each dimension’s score is calculated by dividing the sum of the scores for the responses given to the items in the dimension by the number of the items in that dimension. Higher scores obtained from the overall SBTSN and its dimensions indicate that the individual has more sustainable nutritional behaviors.

Reliability analysis is conducted to test whether the statements on a scale are consistent with each other. In reliability analysis, the Cronbach’s Alpha (*α*) coefficient value ranges between 0 and 1. A value ranging between 0.00 and 0.40 indicates that the scale is unreliable, between 0.41 and 0.60 indicates that the scale has low reliability, between 0.61 and 0.80 indicates that the scale is reliable, and between 0.81 and 1.00 indicates that the scale is highly reliable (27). In the present study, the alpha value was calculated as 0.776 for the Scale for Determining the Minimalist Lifestyle from the Point of Environmental Bioethics, and 0.907 for the Scale for Behaviors toward Sustainable Nutrition. Alpha values for the dimensions ranged between 0.526 and 0.830.

Descriptive statistics regarding the measurement tools were given in Table 2. The mean score was calculated as 85.73 ± 8.96 for the Scale for Determining the Minimalist Lifestyle from the Point of Environmental Bioethics (SDMLPEB) and as 109.76 ± 14.00 for the Scale for Behaviors toward Sustainable Nutrition (SBTSN).

**Table 2:** Descriptive statistics regarding the measurement tools.

Measurement tools	Min.	Max.	x̄	SD
Scale for determining the minimalist lifestyle from the point of environmental bioethics	61	110	85.73	8.96
Minimalist lifestyle	15	34	24.45	3.76
Sustainable environment	32	64	47.86	5.84
Time management	7	19	13.42	2.55
Scale for behaviors toward sustainable nutrition	71	141	109.76	14
Food preference	10	30	21.2	3.52
Food waste reduction	20	45	35.1	5.41
Seasonal and local nutrition	17	40	31.36	4.38
Food purchase	10	30	22.1	3.94

### 2.3 Data collection process

Before the study was conducted, ethical approval was obtained from the Ethics Committee of Çankırı Karatekin University (Decision number: 11, Decision date: February 5, 2024). Since data were collected using the snowball sampling method, the participants were contacted in person, or through email, phone, and digital platforms. The survey was designed using a Google Form for the participants to answer the questions. This method was preferred, because today, the participation rate is higher in studies conducted in a digital environment. The population of the study consisted of individuals aged 18 and above who had economic independence. Individuals aged 18 and above were selected because they are legally considered adults, while those with economic independence were included due to the nature of the research, which requires participants to be capable of determining their own lifestyles. For the research, individuals were informed either face-to-face or by phone. The data collection form link was sent via WhatsApp or email to individuals who agreed to participate in the study. These participants were then asked to forward the research link to individuals around them who met the specified inclusion criteria. It was predicted that the number of participants would be limited if data are collected through traditional methods, namely paper and pencil method. Furthermore, Google Form eliminates the need to collect survey forms physically, which makes it easy to reach individuals in different cities. Only one survey was allowed to be filled out with the same IP in the Google Form. In the first part of the survey form, the purpose of the study was explained to the participants, and they were told that the data collected would be used only for scientific purposes. In this way, informed consent was obtained from the participants regarding the research. The collection of study data was started primarily from the researchers’ immediate circle. They were also asked to send it to their circles. However, it has been determined that the form was also completed by individuals without economic independence, such as students. The responses of these individuals were not included in the study. Of 321 people reached, 283 met the inclusion criteria and filled out the data collection form validly. The data were collected in Turkey, where there is no artificial meat production or sale other than plant-based meats obtained from wheat and legumes such as beans and peas, and products such as falafel, deep-fried balls made from ground fava beans, chickpeas, or both, and mixed with herbs and spices. In other words, it is thought that the majority of individuals in the sample did not consume artificial meat produced with the first two methods mentioned in the literature section. Data was collected between February 23, 2024, and May 26, 2024.

### 2.4 Data analysis

The data obtained in the study were analyzed using the SPSS (Statistical Package for Social Sciences) for Windows, version 25.0. Numbers, arithmetic mean, percentage, standard deviation, and minimum and maximum values were used in the analysis of the descriptive data. Whether the data are normally distributed is tested with Q-Q Plot, or with the skewness and kurtosis values. If the skewness and kurtosis values are within the range of ±3, the data are considered to have normal distribution. In the present study, whether the data were normally distributed was tested with kurtosis and skewness values. All the measurement tools were analyzed for outliers and extreme observations in the data set, and those which were inappropriate were removed from the data set. In normally distributed data, the independent samples t-test was used to determine the difference between two independent groups, ANOVA test was used to determine the difference between more than two independent groups, and Bonferroni multiple comparison test was used to determine from which group(s) the difference stemmed. The relationship between the measurement tools was analyzed with correlation analysis (Pearson correlation analysis). The reason why Pearson correlation analysis is preferred is that the variables included in the study are continuous and the primary aim is to reveal the direction and strength of linear relationships between the variables. The primary aim of the present study was to determine the interrelationships between the variables.

In the present study, the kurtosis and skewness values of all the measurement values were within the range of ±3, indicating that the data were normally distributed. In all the measurements, while the difference between two independent groups was determined with the independent samples t test, the difference between more than two groups was determined with one-way ANOVA (F test). After the ANOVA test, a *Post-hoc* multiple comparison test was performed to identify groups that differed. For the *post hoc* test, Bonferroni was used when variance homogeneity was achieved. If variance homogeneity was not achieved, Tamhane test was used (Table 3).

**Table 3:** Kurtosis and skewness values of the measurement tools.

Measurement tools	Kurtosis	Kurtosis *SE	Skewness	Skewness *SE
Scale for determining the minimalist lifestyle from the point of environmental bioethics	0.315	0.289	−0.145	0.145
Minimalist lifestyle	−0.437	0.289	−0.235	0.145
Sustainable environment	0.055	0.289	−0.211	0.145
Time management	−0.240	0.289	−0.047	0.145
Scale for behaviors toward sustainable nutrition	−0.323	0.289	−0.240	0.145
Food preference	0.007	0.289	−0.282	0.145
Food waste reduction	−0.210	0.289	−0.379	0.145
Seasonal and local nutrition	−0.019	0.289	−0.261	0.145
Food purchase	−0.094	0.289	−0.387	0.145

*SE, Standard Error.

The analysis of data obtained from open-ended questions was conducted in accordance with the thematic analysis proposed by Braun and Clarke (28). These stages include familiarization with the data, generating initial codes, identifying themes, reviewing themes, defining and naming themes, and producing the final report. An inductive approach was adopted during the interpretation process, and the data were examined to identify meaningful codes aligned with the research questions. The data were organized through Open Coding. In the final stage, themes were constructed from the identified codes.

### 2.5 Limitations

Potential sampling bias arises due to the homogeneity of socio-economic groups and reliance on network-driven recruitment. This bias limits the generalizability of the findings to the wider population. The results were interpreted by considering the characteristics of the study sample. Since the snowball (chain) sampling method was employed, it is believed that the participants predominantly come from certain socio-economic backgrounds and possess an intellectual perspective.

## 3 Results

Because the study sample was determined using the snowball sampling method, the results reflect the views of a certain group. Therefore, generalizing the results to the general population is methodologically not feasible. The present study was conducted with individuals who were over 18 years of age and had economic independence. Their demographic characteristics are given under the heading “Population and Sample of the Study.” Other characteristics of the participants were given in Table 4.

**Table 4:** Distribution of the participants according to their other characteristics.

Characteristics		*n*	%
Type of foods mostly consumed in the diet*	Meat, vegetables and grains in equal amounts	201	71
	Rich in vegetables and grains	63	22.3
	Rich in meat	45	15.9
	Fast food, ready-made food (hamburgers. French fries. Pizza etc.)	12	4.2
	Rich in sausage, salami	6	2.1
	Vegetarian	4	1.4
	Vegan	2	0.7
Do you have an animal regularly looked after in the house/garden?	Yes	96	33.9
	No	187	66.1

*Some of the participants chose more than one answer.

The analysis of the other characteristics of the participants revealed that the majority (71%) of them had a diet consisting of meat, vegetables and grains in equal amounts, 22.3% had a diet mainly consisting of vegetables and grains, 15.9% had a diet mainly consisting of meat, and 66.1% of them did not have an animal such as a cat or dog that they regularly looked after at home or in their garden (Table 4).

Responses given by the participants revealed that, of them, the majority (65%) were not knowledgeable about artificial meat, a great majority (83.4%) did not know how artificial meat was produced, most (87.6%) would not prefer artificial meat, and half (51.6%) did not and would not have a positive perspective of artificial meat. On the other hand, of the participants 27.2 and 26.9% had a positive view about artificial meat because they think that it is environmentally sustainable and it will lessen animal use, respectively (Table 5).

**Table 5:** Distribution of the participants according to their views about artificial meat.

Views		*n*	%
Are you knowledgeable about artificial meat	Yes	99	35
	No	184	65
Do you know how artificial meat is produced?	Yes	47	16.6
	No	236	83.4
Would you consume artificial meat?	Yes	35	12.4
	No	248	87.6
Do you think you would all consume artificial meat in your family?	Yes	29	10.2
	No	254	89.8
What are the factors causing you to have a positive or more positive view about artificial meat? *	There can be no positive perspective	146	51.6
	Environmentally sustainable	77	27.2
	Less animal use	76	26.9
	Economical	67	23.7
	Ease of access	37	13.1
	Other	17	6

*Some of the participants chose more than one answer.

The relationship between the measurement tools was analyzed by Pearson correlation analysis. The results of the correlation analysis revealed the following: There were statistically significant positive correlations between the score obtained from the Minimalist Lifestyle dimension of the Scale for Determining the Minimalist Lifestyle from the Point of Environmental Bioethics (SDMLPEB) and the mean scores obtained from the Food Preference (r = 0.162; *p <* 0.05), Food Waste Reduction (r = 0.178; *p <* 0.05), Seasonal and Local Nutrition (r = 0.242; *p <* 0.05) and Food Purchase (r = 0.125; *p <* 0.05) dimensions of the Scale for Behaviors toward Sustainable Nutrition (SBTSN). There was a statistically significant positive correlation between the score obtained from the Minimalist Lifestyle dimension of the SDMLPEB and the mean score obtained from the SBTSN (r = 0.220; *p <* 0.05) (Table 6).

**Table 6:** Relationship between the measurement tools.

Scales and their dimensions		1	2	3	4	5	6	7	8	9
1. Minimalist Lifestyle	*r*	1								
	*p*									
2. Sustainable Environment	*r*	0.242^**^	1							
	*p*	0.000								
3. Time Management	*r*	0.341^**^	0.282^**^	1						
	*p*	0.000	0.000							
Scale for Determining the Minimalist Lifestyle from the Point of Environmental Bioethics	*r*	0.674^**^	0.834^**^	0.611^**^	1					
	*p*	0.000	0.000	0.000						
1. Food Preference	*r*	0.162^**^	0.372^**^	0.166^**^	0.358^**^	1				
	*p*	0.006	0.000	0.005	0.000					
2. Food Waste Reduction and Food Purchase	*r*	0.178^**^	0.582^**^	0.187^**^	0.507^**^	0.456^**^	1			
	*p*	0.003	0.000	0.002	0.000	0.000				
3. Seasonal and Local Nutrition	*r*	0.242^**^	0.373^**^	0.151^*^	0.388^**^	0.525^**^	0.552^**^	1		
	*p*	0.000	0.000	0.011	0.000	0.000	0.000			
4. Food Purchase	*r*	0.125^*^	0.436^**^	0.187^**^	0.390^**^	0.597^**^	0.555^**^	0.573^**^	1	
	*p*	0.035	0.000	0.002	0.000	0.000	0.000	0.000		
Scale for Behaviors toward Sustainable Nutrition	*r*	0.220^**^	0.558^**^	0.214^**^	0.517^**^	0.760^**^	0.830^**^	0.819^**^	0.825^**^	1
	*p*	0.000	0.000	0.000	0.000	0.000	0.000	0.000	0.000	

**p < 0.01; *p < 0.05.

There were statistically significant positive correlations between the score obtained from the Sustainable Environment dimension of the SDMLPEB and the mean scores obtained from the Food Preference (r = 0.372; *p <* 0.05), Food Waste Reduction (r = 0.582; *p <* 0.05), Seasonal and Local Nutrition (r = 0.373; *p <* 0.05) and Food Purchase (r = 0.436; *p <* 0.05) dimensions of the SBTSN. There was a statistically significant positive correlation between the score obtained from the Sustainable Environment dimension of the SDMLPEB and the mean score obtained from the SBTSN (r = 0.558; *p <* 0.05) (Table 6).

There were statistically significant positive correlations between the score obtained from the Time Management dimension of the SDMLPEB and the mean scores obtained from the Food Preference (r = 0.166; *p <* 0.05), Food Waste Reduction (r = 0.187; *p <* 0.05), Seasonal and Local Nutrition (r = 0.151; *p <* 0.05) and Food Purchase (r = 0.187; *p <* 0.05) dimensions of the SBTSN. There was a statistically significant positive correlation between the score obtained from the Time Management dimension of the SDMLPEB and the mean score obtained from the SBTSN (r = 0.214; *p <* 0.05) (Table 6).

There were statistically significant positive correlations between the score obtained from the SDMLPEB and the mean scores obtained from the Food Preference (r = 0.358; *p <* 0.05), Food Waste Reduction (r = 0.507; *p <* 0.05), Seasonal and Local Nutrition (r = 0.388; *p <* 0.05) and Food Purchase (r = 0.390; *p <* 0.05) dimensions of the SBTSN. There was a statistically significant positive correlation between the score obtained from the SDMLPEB and the mean score obtained from the SBTSN (r = 0.517; *p <* 0.05).

The comparison of the scores obtained from the SDMLPEB and its dimensions according to demographic characteristics of the participants demonstrated that there were statistically significant differences between the participants’ scores according to the age variable (*p <* 0.05). In this study, the results obtained based on the participants’ descriptive characteristics were also evaluated. Accordingly, only variables found to be statistically significant are included in the article.

The multiple comparison test conducted to identify from which group the difference stemmed revealed that the difference stemmed from the scores of the participants aged ≥56 years. They obtained higher scores from the Sustainable Environment dimension than did the participants aged ≤35 years and those aged between 36 and 45 years. These results suggest that the participants aged ≥56 years adopted an environmentally sustainable life more, and that the participants aged ≤35 years adopted a lifestyle very different from the environmentally sustainable lifestyle (Table 7).

**Table 7:** Comparison of the scores obtained from dimensions of the scale for determining the minimalist lifestyle from the point of environmental bioethics.

Characteristics		Minimalist lifestyle	Sustainable environment	Time management	Scale for determining the minimalist lifestyle from the point of environmental bioethics
		Mean ± SD	Mean ± SD	Mean ± SD	Mean ± SD
1. Age	<= 35.00 (1)	23.97 ± 4.03	45.98 ± 6.48	13.1 ± 2.61	83.05 ± 9.59
	36.00–45.00 (2)	24.57 ± 3.53	46.49 ± 4.86	12.97 ± 2.78	84.03 ± 8.4
	46.00–55.00 (3)	25.11 ± 3.83	48.45 ± 6.19	14.02 ± 2.5	87.57 ± 9.6
	56.00 + (4)	24.17 ± 3.68	49.88 ± 5.2	13.58 ± 2.26	87.64 ± 7.78
***F*-value**		1.176	7.534	2.386	5.079
***p*-value**		0.319	0.000*	0.069	0.002*
***Post-hoc***			4 > 1.2		1 < 3.4
2. Sex	Women	24.47 ± 3.86	47.53 ± 5.89	13.48 ± 2.53	85.48 ± 8.98
	Men	24.38 ± 3.5	48.71 ± 5.68	13.28 ± 2.61	86.36 ± 8.95
***t*-value**		0.197	−1.540	0.617	−0.743
***p*-value**		0.844	0.125	0.538	0.458
3. Marital status	Married	24.57 ± 3.81	47.97 ± 5.73	13.29 ± 2.6	85.83 ± 8.97
	Single	24.11 ± 3.61	47.59 ± 6.16	13.76 ± 2.4	85.47 ± 8.98
***t*-value**		0.923	0.478	−1.380	0.307
***p*-value**		0.357	0.633	0.169	0.759
4. Having children	Yes	24.51 ± 3.79	47.91 ± 5.49	13.32 ± 2.5	85.74 ± 8.41
	No	24.29 ± 3.69	47.76 ± 6.66	13.67 ± 2.66	85.72 ± 10.23
***t*-value**		0.450	0.191	−1.066	0.010
***p*-value**		0.653	0.849	0.287	0.992
5. The number of the children	1	24.4 ± 4.05	47.14 ± 5.56	13.22 ± 2.65	84.75 ± 8.84
	2	24.48 ± 3.44	48.53 ± 5.49	13.3 ± 2.38	86.31 ± 8.18
	3	25 ± 4.44	49.12 ± 5.44	13.65 ± 2.47	87.76 ± 7.81
***F*-value**		0.179	1.861	0.211	1.304
***p*-value**		0.836	0.158	0.810	0.274
6. Educational status	Senior high school and lower	25.57 ± 4.59	49.64 ± 6.74	13 ± 1.96	88.21 ± 10.17
	University	24.7 ± 3.54	48.31 ± 5.59	13.54 ± 2.51	86.55 ± 8.49
	Master’s degree	24.05 ± 3.86	47.19 ± 5.97	13.34 ± 2.65	84.57 ± 9.24
***F*-value**		1.687	1.932	0.412	2.216
***p*-value**		0.187	0.147	0.663	0.111
7. Profession	Academician (1)	24.04 ± 3.16	47.07 ± 6.29	12.96 ± 2.54	84.07 ± 8.74
	Physician (2)	23.74 ± 5.11	47.35 ± 6.23	13.45 ± 2.98	84.55 ± 10.15
	Nurse (3)	24.69 ± 3.59	46.81 ± 5.74	12.81 ± 2.74	84.31 ± 8.91
	Teacher (4)	24.28 ± 3.61	47.41 ± 4.26	13.76 ± 2.1	85.45 ± 6.13
	Civil Servant (5)	25.33 ± 2.81	49.33 ± 6.92	13.89 ± 2.17	88.56 ± 8.89
	Engineer (6)	24.41 ± 3.37	48.19 ± 5.88	13.41 ± 2.68	86 ± 8.75
	Retired (7)	25.61 ± 3.3	50.29 ± 4.75	14.46 ± 2.27	90.36 ± 6.79
	Self-employed (8)	23.79 ± 3.93	46.63 ± 6.13	13.04 ± 2.51	83.46 ± 9.49
	Other (9)	25.34 ± 4.16	49.25 ± 5.3	13.59 ± 2.7	88.19 ± 9.9
***F*-value**	1.148	1.508	1.216	2.277	
***p*-value**	0.339	0.154	0.289	0.023*	
***Post-hoc***				7 > 8	
8. Perceived income status	Good (1)	24.83 ± 3.84	47.53 ± 6.08	13.79 ± 2.76	86.15 ± 9.27
	Moderate (2)	24.26 ± 3.66	48.14 ± 5.74	13.36 ± 2.31	85.75 ± 8.72
	Not good (3)	23.29 ± 3.95	47.64 ± 5.14	11.07 ± 2.06	82 ± 8.75
***F*-value**	1.475	0.367	7.592	1.347	
***p*-value**	0.231	0.693	0.001*	0.262	
***Post-hoc***			3 < 1.2		
10. Do you have an animal regularly looked after in the house/garden?	Yes	23.98 ± 3.97	48.11 ± 6.06	13.79 ± 2.6	85.89 ± 9.21
	No	24.68 ± 3.63	47.73 ± 5.74	13.24 ± 2.51	85.65 ± 8.85
***F*-value**	−1.499	0.520	1.744	0.207	
***p*-value**	0.135	0.604	0.082	0.836	
11. Are you knowledgeable about artificial meat	Yes	25.07 ± 3.8	48.14 ± 5.77	13.82 ± 2.54	87.03 ± 8.91
	No	24.11 ± 3.7	47.71 ± 5.9	13.21 ± 2.54	85.03 ± 8.94
***t*-value**	2.067	0.589	1.917	1.795	
***p*-value**	0.040*	0.556	0.056	0.074	
12. Do you know how artificial meat is produced?	Yes	25.04 ± 3.49	48.04 ± 5.49	13.62 ± 2.9	86.7 ± 8.49
	No	24.33 ± 3.8	47.83 ± 5.92	13.39 ± 2.48	85.54 ± 9.06
***t*-value**	1.195	0.231	0.568	0.813	
***p*-value**	0.233	0.817	0.571	0.417	
14. Would you consume artificial meat?	Yes	22.6 ± 3.92	46.71 ± 6.11	13.2 ± 2.18	82.51 ± 9.3
	No	24.71 ± 3.67	48.02 ± 5.8	13.46 ± 2.6	86.19 ± 8.84
***t*-value**	−3.154	−1.243	−0.555	−2.285	
***p*-value**	0.002*	0.215	0.580	0.023*	
15. Do you think you would all consume artificial meat in your family?	Yes	23.66 ± 3.73	47.76 ± 4.88	13.38 ± 2.13	84.79 ± 7.91
	No	24.54 ± 3.76	47.87 ± 5.95	13.43 ± 2.6	85.84 ± 9.08
***t*-value**	−1.197	−0.101	−0.100	−0.594	
***p*-value**	0.232	0.920	0.921	0.553	

*p < 0.05. t: Independent Samples *T-*Test; F, One-Way Analysis of Variance.

The comparison of the scores the participants obtained from the dimensions of the SDMLPEB in terms of their professions demonstrated that there was not a statistically significant difference between them (*p* > 0.05). However, according to the comparison of the scores they obtained from the overall SDMLPEB, there was a statistically significant difference between them. The retired participants obtained higher scores than the self-employed ones. Since the vast majority of the retired participants were ≥56 years old, this result was an expected result. The scores they obtained from the time management dimension of the SDMLPEB differed statistically significantly according to the participants’ demographic characteristics. Of them, those who perceived their income as not good obtained lower scores than did those who perceived their income as good or moderate.

The scores they obtained from the Minimalist Lifestyle dimension of the SDMLPEB also differed statistically significantly according to the participants’ knowledge about artificial meat variable (*p <* 0.05). Of them, those who were knowledgeable about artificial meat obtained higher scores than did those who were not.

The scores they obtained from the SDMLPEB and its Minimalist Lifestyle dimension also differed statistically significantly according to the participants’ preference to consume artificial meat variable (*p <* 0.05). Those who adopted a Minimalist Lifestyle were more likely not to prefer to consume artificial meat (Table 8).

**Table 8:** Comparison of the scores obtained from the scale for behaviors toward sustainable nutrition tools according to demographic characteristics.

Characteristics	Food preference	Food waste reduction	Seasonal and local nutrition	Food purchase	Scale for behaviors toward sustainable nutrition
	Mean ± SD	Mean ± SD	Mean ± SD	Mean ± SD	Mean ± SD
1. Age					
<= 35.00 (1)	20.08 ± 3.4	33.92 ± 5.42	29.45 ± 4.49	20.9 ± 3.54	104.35 ± 12.99
36.00–45.00 (2)	20.44 ± 3.75	33.35 ± 4.62	31 ± 3.8	21.71 ± 4.2	106.5 ± 12.83
46.00–55.00 (3)	21.34 ± 3.45	35.68 ± 5.57	31.65 ± 4.3	22.25 ± 3.5	110.91 ± 13.23
56.00 + (4)	22.52 ± 3.03	36.94 ± 5.3	32.78 ± 4.36	23.16 ± 4.07	115.41 ± 14.18
***p*-value**	0.000*	0.000*	0.000*	0.005*	0.000*
***Post-hoc***	1.2 < 4	1.2 < 4	1 < 4.5	1 < 4	1 < 4.5
2. Sex					
Women	21.41 ± 3.43	35.36 ± 5.33	31.55 ± 4.28	22.33 ± 3.89	110.66 ± 13.67
Men	20.69 ± 3.71	34.41 ± 5.57	30.89 ± 4.62	21.51 ± 4.03	107.5 ± 14.65
***t*-value**	1.557	1.336	1.141	1.586	1.713
***p*-value**	0.121	0.183	0.255	0.114	0.088
3. Marital status					
Married	21.34 ± 3.44	35.26 ± 5.48	31.76 ± 4.33	22.21 ± 4.08	110.57 ± 14.24
Single	20.85 ± 3.71	34.67 ± 5.23	30.33 ± 4.36	21.84 ± 3.57	107.68 ± 13.21
***t*-value**	1.062	0.822	2.487	0.709	1.559
***p*-value**	0.289	0.412	0.013*	0.479	0.120
4. Having children?					
Yes	21.46 ± 3.43	35.29 ± 5.47	31.83 ± 4.25	22.26 ± 4.01	110.83 ± 13.83
No	20.59 ± 3.67	34.63 ± 5.25	30.24 ± 4.52	21.73 ± 3.76	107.19 ± 14.15
***t*-value**	1.901	0.940	2.803	1.011	2.000
***p*-value**	0.058	0.348	0.005*	0.313	0.046*
5. The number of the children					
1	20.98 ± 3.56	34.83 ± 5.74	31.88 ± 4.27	22.15 ± 3.99	109.83 ± 13.86
2	21.88 ± 3.14	35.96 ± 5.12	31.99 ± 4.15	22.66 ± 3.88	112.49 ± 13.4
3	21.35 ± 4.23	34.53 ± 5.65	31.29 ± 4.04	20.47 ± 4.6	107.65 ± 14.98
***F*-value**	1.589	1.180	0.198	2.213	1.372
***p*-value**	0.207	0.309	0.821	0.112	0.256
6. Educational status					
Senior high school and lower (1)	20.5 ± 4.52	32.93 ± 6.29	29.86 ± 5.59	21.36 ± 3.86	104.64 ± 16.37
University (2)	21.53 ± 3.61	35.99 ± 5.45	32.07 ± 4.51	22.77 ± 3.82	112.36 ± 14.43
Master’s degree (3)	20.93 ± 3.29	34.36 ± 5.12	30.75 ± 3.98	21.46 ± 3.99	107.5 ± 12.76
***F*-value**	1.268	4.359	3.996	4.085	5.194
***p*-value**	0.283	0.014*	0.019*	0.018*	0.006*
***Post-hoc***		2 > 3	2 > 3	2 > 3	2 > 3
7. Profession					
Academician (1)	20.89 ± 3.17	34.52 ± 5.42	31.02 ± 3.57	22.41 ± 3.78	108.83 ± 12.65
Physician (2)	20.77 ± 3.02	34 ± 5.16	30.35 ± 3.55	20.9 ± 3.88	106.03 ± 11.41
Nurse (3)	22.06 ± 2.79	35.69 ± 3.42	31.69 ± 4.06	21.94 ± 4.46	111.38 ± 11.81
Teacher (4)	22.17 ± 4.17	35.59 ± 5.12	31.62 ± 5.22	22.72 ± 3.63	112.1 ± 15.04
Civil Servant (5)	20.94 ± 4.92	34.67 ± 6.59	31.33 ± 5.46	21 ± 4.74	107.94 ± 19.34
Engineer (6)	20.93 ± 3.17	35.56 ± 5.85	32.04 ± 4.54	21.81 ± 3.62	110.33 ± 14.87
Retired (7)	22.32 ± 3.27	37.18 ± 3.99	33.07 ± 4.6	23.89 ± 3.67	116.46 ± 13.26
Self-employed (8)	19.9 ± 3.55	34.19 ± 6.28	30.63 ± 4.72	21.17 ± 3.97	105.88 ± 14.33
Other (9)	22.22 ± 3.24	35.78 ± 5.12	31.56 ± 4.1	22.97 ± 3.74	112.53 ± 12.68
***F*-value**	2.119	1.084	1.063	1.997	1.948
***p*-value**	0.034*	0.374	0.389	0.047	0.053
***Post-hoc***	7 > 8				
8. Perceived income status					
Good (1)	20.98 ± 3.46	35.34 ± 5.16	31.62 ± 4.23	21.71 ± 3.89	109.66 ± 13.19
Moderate (2)	21.49 ± 3.45	35.02 ± 5.54	31.39 ± 4.32	22.57 ± 3.86	110.47 ± 14.11
Not good (3)	20 ± 4.57	33.86 ± 6.18	28.86 ± 5.65	20.29 ± 4.53	103 ± 18.21
***F*-value**	1.547	0.502	2.528	3.200	1.840
***p*-value**	0.215	0.606	0.082	0.042*	0.161
***Post-hoc***				2 > 3	
10. Do you have an animal regularly looked after in the house/garden?					
Yes	21.83 ± 3.17	34.97 ± 5.57	31.11 ± 4.66	22.47 ± 4.11	110.39 ± 14.49
No	20.88 ± 3.65	35.16 ± 5.33	31.49 ± 4.24	21.91 ± 3.85	109.44 ± 13.77
***t*-value**	2.166	−0.282	−0.676	1.121	0.535
***p*-value**	0.031	0.778	0.500	0.263	0.593
11. Are you knowledgeable about artificial meat?					
Yes	21.66 ± 3.43	35.38 ± 5.42	31.41 ± 4.06	22.44 ± 4.2	110.9 ± 14
No	20.96 ± 3.55	34.94 ± 5.41	31.33 ± 4.55	21.92 ± 3.79	109.15 ± 14
***t*-value**	1.588	0.658	0.151	1.071	1.001
***p*-value**	0.113	0.511	0.880	0.285	0.318
12. Do you know how artificial meat is produced?					
Yes	21.11 ± 4.01	35.6 ± 5.18	31.04 ± 3.82	21.7 ± 3.98	109.45 ± 13.32
No	21.22 ± 3.42	35 ± 5.46	31.42 ± 4.49	22.18 ± 3.94	109.83 ± 14.16
***t*-value**	−0.210	0.694	−0.544	−0.762	−0.169
***p*-value**	0.834	0.488	0.587	0.447	0.866
14. Would you consume artificial meat?					
Yes	20.31 ± 3.21	34.17 ± 6.2	28.46 ± 4.37	21.2 ± 3.83	104.14 ± 14.55
No	21.33 ± 3.55	35.23 ± 5.29	31.77 ± 4.23	22.23 ± 3.95	110.56 ± 13.77
***t*-value**	−1.604	−1.080	−4.318	−1.450	−2.562
***p*-value**	0.110	0.281	0.000*	0.148	0.011*
15. Do you think you would all consume artificial meat in your family?					
Yes	21.07 ± 2.8	35.21 ± 5.85	29.52 ± 4.76	22.00 ± 3.72	107.79 ± 15.28
No	21.22 ± 3.6	35.08 ± 5.37	31.57 ± 4.3	22.11 ± 3.97	109.99 ± 13.86
***t*-value**	−0.219	0.117	−2.412	−0.148	−0.799
***p*-value**	0.827	0.907	0.017*	0.883	0.425

*p < 0.05. t: Independent Samples *T-*Test; F, One-Way Analysis of Variance.

There were statistically significant differences between the participants in terms of the scores they obtained from the Scale for Behaviors toward Sustainable Nutrition (SBTSN), and its Food Preference, Food Waste Reduction, Seasonal and Local Nutrition and Food Purchase dimensions in terms of the age variable (*p <* 0.05). According to the multiple comparison test conducted to identify from which group the difference stemmed, the participants aged ≤35 years obtained lower scores from the Food Preference and Food Waste Reduction dimensions than did the participants aged between 36 and 45 years and the participants aged ≥56 years. The participants aged ≤35 years obtained lower scores from the Seasonal and Local Nutrition dimension than did those aged ≥56 years and those between the ages of 46 and 55 years. The participants aged ≤35 years also obtained lower scores from the Food Purchase dimension than did those aged ≥56 years. As for the overall SBTSN, the participants aged ≤35 years also obtained lower scores than did those between the ages of 46 and 55 years and those aged ≥56 years. All these results indicate that the participants aged ≤35 years generally received lower scores from the overall SBTSN and its dimensions than did the other participants. These results are consistent with those obtained from the Scale for Determining the Minimalist Lifestyle from the Point of Environmental Bioethics (SDMLPEB).

There were also statistically significant differences between the scores the participants obtained from the Seasonal and Local Nutrition dimension according to the marital status variable (*p <* 0.05). The participants who were married obtained higher scores than did those who were single. There were also statistically significant differences between the scores the participants obtained from the SBTSN and its Seasonal and Local Nutrition dimension according to the having children variable (*p <* 0.05). Those who had children obtained higher scores than did those who did not have children (Table 8). It is thought that these results are interconnected since the married participants had children and therefore their Seasonal and Local Nutrition scores were higher.

There were also statistically significant differences between the scores the participants obtained from the SBTSN and its Food Waste Reduction, Seasonal and Local Nutrition and Food Purchase dimensions in terms of the educational status variable (*p <* 0.05). According to the multiple comparison test conducted to identify from which group the difference stemmed, the university graduate participants obtained higher scores than did the participants with the master’s degree (Table 8).

There were also statistically significant differences between the scores the participants obtained from the Food Preference dimension of the SBTSN in terms of the profession variable (*p <* 0.05). The retired participants obtained higher scores than the self-employed participants did. There were also statistically significant differences between the scores the participants obtained from the Food Purchase dimension in terms of the perceived income status variable. The participants with poor income obtained lower scores than did the participants with moderate income (Table 8). Considering that the economic levels of individuals will have a direct effect on their food purchase, this was not an expected result.

There were also statistically significant differences between the scores the participants obtained from the overall SBTSN and its Seasonal and Local Nutrition dimension in terms of preferring to consume artificial meat variable (*p <* 0.05). The participants who preferred to consume artificial meat obtained lower scores than did the participants who did not, which suggests that for individuals who prefer to consume artificial meat, it is not very important to eat healthy foods by consuming seasonal and local foods. However, contrary to this result, the participants who consume artificial meat in their families obtained higher scores from the Seasonal and Local Nutrition dimension than did the participants who would not consume artificial meat in their families (*p <* 0.05, Table 8). This result indicates that sustainable nutrition was the common goal among the participants.

The distribution of the participants in terms of their opinions on artificial meat was given in Tables 8, 9. The three statements most frequently made by the participants regarding whether artificial meat production would be beneficial or harmful are as follows: “harmful to health (36.0%),” “I do not know (18.0%),” “harmful (15.0%)” and “beneficial for environmental sustainability (6.5%)” (Table 9).

**Table 9:** Distribution of the responses the participants gave to the question “do you think artificial meat production will be beneficial or harmful?”

Responses*	*n*	%
Harmful to health	85	36.0
I have no idea	43	18.0
Harmful	35	15.0
Beneficial in terms of environmental sustainability	15	6.5
Beneficial	9	4.0
Good in terms of animal rights	7	3.0
It causes environmental problems. It harms the environment	6	2.5
I am undecided	6	2.5
It can be consumed only by being careful	6	2.5
We do not know its long-term consequences	5	2.0
Economical	5	2.0
It is both beneficial and harmful	4	1.5
others	11	4.5
Total	237	100.0

*While some of the participants gave more than one answer, some did not answer.

Some of the participants’ opinions on the subject are given below. Their responses indicated that although they thought that artificial meat production would be good because it would ensure environmental sustainability and decrease environmental problems, they still had some doubts in terms of human health:

“It will be positive in terms of environmental and animal sensitivity. However, I am not sure if it will have the same nutritional value as real meat.” (Woman, 54 years old, academician)

“...Its being sustainable is important. Human health is important. I don’t think those produced in the market could be as reliable as those produced in a laboratory environment” (Woman, 56 years old, pharmacist)

“I think it could be beneficial in terms of environmental sustainability, but I am not sure whether the consumer will receive enough natural nutrients.” (Woman, 49 years old, physician)

“I think artificial meat is against human nature. Even though it is necessary for the environment, I would not prefer to consume a product that is artificial; I would prefer not to eat meat at all.” (Woman, 47 years old, pharmacist)

“I think it will be an indispensable reality for a sustainable environment and economy-related issues that we will inevitably face in the future.” (Woman, 50 years old, consultant)

The three statements most frequently made by the participants regarding their opinions about artificial meat are as follows: “unhealthy (28.0%),” “I have no idea (18.0%)” and “negative (10.0%).” While some participants had no idea about the subject, some participants were knowledgeable enough (Table 10):

“It is something I have never thought about. Thanks to your research, I have done some reading. It could contribute to the environment positively.” (Woman, 54 years old, faculty member)

“If the problem is animal consumption and methane gas, the solution to this problem is not artificial meat, but controlled consumption and the closure of consumption-oriented farms in developed countries. The solution to their luxury consumption should not be paid by developing countries. In addition, even if artificial meat consumption becomes widespread, natural farms for the privileged classes will continue and, as always, innocent people will try to fix things. Moreover, the artificial meat sector will cause the creation of new needs and will enrich some people” (Woman, 29 years old, editor)

Some of the participants stated that they would consume artificial meat if they had to or because they would have to, even if they did not prefer to do so.

**Table 10:** Distribution of the responses the participants gave to the question “what do you think about artificial meat?”

Responses*	*n*	%
Unhealthy	74	28.0
I have no idea	47	18.0
Negative	26	10.0
I do not prefer to consume ir / It should not be consumed	20	7.5
Harmful	15	5.5
Beneficial in terms of environmental sustainability	12	4.5
I am undecided	9	3.5
Unnecessary	7	2.5
It can be consumed only by being careful	7	2.5
Positive	7	2.5
It may be the last resort	7	2.5
It is not a substitute for real meat. (It cannot substitute for real meat.)	5	2.0
Harmful to the ecosystem	4	1.5
Economical	4	1.5
I want to try it	4	1.5
Plant-based artificial meats can be preferred	3	1.0
We do not know its long-term consequences	1	0.5
Good in terms of animal rights	1	0.5
Others	12	4.5
Total	265	100.0

*While some of the participants gave more than one answer, some did not answer.

“It may contribute to my environmental and ethical responsibilities. I do not prefer to consume it, but if the conditions require it and there is no other option, I may choose to consume it.” (Man, 40 years old, academician)

“In a world where the human population is increasing very rapidly, if artificial meat helps fight against hunger, if it can be produced in a truly healthy way and if it is economical, why not … (Man, 60 years old, captain)

“We have to. Even vegetables and fruits will be artificial. The world's resources are depleted. The population is very high. There is no other way. But I don't eat it unless I have to.” (Woman, 60 years old, retired bank manager)

Artificial meat production is considered not only as a way to enhance ecological sustainability but also as a potential protein alternative to meet the needs of an expanding world population (5). Some of the participants expressed their view on this issue as follows:

“It could be a solution for the lower income group who cannot afford protein.” (Man, 58 years old, faculty member)

“In the future, artificial meat production may meet people’s protein consumption needs and minimize the harm caused to animals…” (Woman, 58 years old, teacher)

The following statements are examples of the opinions of the participants who approached to the issue from different aspects:

“I have been a vegetarian for a very long time. I am sensitive about the connection between living beings and meat, so the word meat especially bothers me.” (Woman, 49 years old, academic artist)

“Whether the situation of producers who make a living from animal husbandry will become another economic problem will be understood in time.” (Man, 68 years old, dentist)

The opinions of vegan/vegetarian participants were also observed. A vegan participant thought that artificial meat production was good in terms of environmental sustainability and less animal use. She also stated that it was suitable for vegan consumption. A participant who was neither a vegan nor a vegetarian stated the following:

“Although I am neither a vegan nor a vegetarian, I do not consume meat often. However, I am aware that meat consumption is very high worldwide. If artificial meat production does not cause environmental pollution during the production phase and does not cause additional harm to the human body, why not?” (Woman, 27 years old, Software Developer)

## 4 Discussion

The data obtained revealed that there were statistically significant positive relationships between the scores obtained from the overall Scale for Determining the Minimalist Lifestyle from the Point of Environmental Bioethics and its Minimalist Lifestyle, Sustainable Environment and Time Management dimensions and the scores obtained from overall the Scale for Behaviors toward Sustainable Nutrition and its dimensions and its dimensions.

In other words, there was a statistically significant positive relationship between the scores obtained from the two scales and their dimensions. People who adopt a minimalist lifestyle also behave in accordance with sustainable nutrition. It is an expected result that individuals who were aware of environmental sustainability obtained high scores from both scales. These results suggest that the Minimalist Lifestyle also has an environmentally sustainable feature, which is supported by several studies in the literature (29, 30). The comparison of the scores obtained from the overall Scale for Determining the Minimalist Lifestyle from the Point of Environmental Bioethics (SDMLPEB) and its dimensions in terms of the participants’ demographic characteristics demonstrated that there was a statistically significant difference between the participants’ scores according to the age variable. The participants aged ≥56 years obtained higher scores from the Sustainable Environment dimension than did those aged ≤35 years and those aged between 36 and 45 years. The participants aged ≤35 years obtained lower scores from the overall SDMLPEB than did those between the ages of 46 and 55 and those aged ≥56 years, suggesting that the participants who were older adopted a minimalist lifestyle more. This is probably because older adults based on their life experiences think that belongings or purchased goods are not very important.

The retired participants obtained higher scores from the overall SDMLPEB than did the self-employed participants, which is probably due the fact that retired participants were a little older and therefore they had a greater number of life experiences.

The analysis of the data on artificial meat revealed that those who said they were knowledgeable about artificial meat obtained higher scores from the Minimalist Lifestyle dimension, which suggests that those who adopted a minimalist lifestyle were curious about the subject of artificial meat and read something about it. However, being knowledgeable about the subject does not mean that it will be adopted. In fact, those who did not prefer to consume artificial meat obtained higher scores from the overall SDMLPEB than did those who did. Thus, it can be inferred that the reason for preferring to consume artificial meat was not associated with the minimalist lifestyle, and that other reasons may have affected their preference. The minimalist lifestyle represents an approach that aims to simplify life, avoid unnecessary consumption, and live a life more in harmony with nature. In this context, the preference for natural foods and a diet based on vegetables and grains can be considered a natural extension of minimalist values. From this perspective, it is understandable that individuals who embrace a minimalist lifestyle are reluctant to consume artificial meat. Artificial meat is a technology developed primarily to allow people to maintain their meat consumption habits. This is a formal change, rather than a reduction, in meat consumption. Individuals who embrace a minimalist lifestyle, however, may advocate for a substantive, rather than formal, reduction in consumption. Therefore, artificial meat products may not align with their values and could be one of the reasons for their reluctance to consume them.

Furthermore some of the participants’ views about artificial meat were not in terms of environmental sustainability, but in terms of the increasing world population and alternative protein sources.

The scores the participants obtained from the dimensions of the Scale for Behaviors toward Sustainable Nutrition (SBTSN) differed according to the age variable. The participants aged ≤35 years obtained lower scores from the Food Preference and Food Waste Reduction dimensions than did those aged between 36 and 45 years and those aged ≥56 years, from the Seasonal and Local Nutrition sub-dimension than did those aged ≥56 years and those aged between 46 and 55 years, from the Food Purchase dimension than did those aged ≥56 years, and from the overall SBTSN than did those aged between 46 and 55 years and those aged ≥56 years.

One of the factors that affect people’s food preferences may be health problems they have with increasing age. It is thought that these health problems may have affected the results obtained, and that advanced age and experience are effective in preventing waste.

There was a statistically significant difference between the scores the participants obtained from the Seasonal and Local Nutrition dimension of the scale according to the marital status variable (*p <* 0.05). The married participants obtained higher scores than did the single participants. There were also statistically significant differences between the scores the participants obtained from the overall SBTSN and its Seasonal and Local Nutrition dimension according to the having children variable (*p <* 0.05). The participants who had children obtained higher scores than did those who did not have children (Table 8). The factor affecting their having higher scores was probably due to their wish that their child eats healthy foods.

The scores the participants obtained from the three dimensions of the SBTSN varied according to the educational status variable. The results of the statistical tests demonstrated that the participants who were university graduates obtained higher scores than did those with master’s degree. The scores obtained from the Food Purchase dimension were directly proportional to the income status of the participants, indicating that sustainable nutrition behavior is dependent not only on the development of awareness about the subject, but also on the financial status.

The scores the participants obtained from overall the SBTSN and its Seasonal and Local Nutrition dimensions varied according to the preferring to consume artificial meat variable. Those who preferred to consume artificial meat obtained lower scores than did those who did not. The scores the participants obtained from overall the Seasonal and Local Nutrition dimension varied according to the preferring to consume artificial meat in the family variable. Those who preferred to consume artificial meat in the family obtained higher scores than did those who did not prefer to consume artificial meat in the family. The different results between the scores obtained from the scale and artificial meat consumption can be attributed to the concerns of individuals about artificial meat, because according to the research, most of the participants did not think that artificial meat was good for their health and therefore they approached it cautiously.

When approached from the cultural context of Turkish society; Meat consumption occupies a central place in Turkey’s traditional dietary culture. According to the perception of some individuals, a meal without meat is considered neither sufficiently satisfying nor of high quality. In the culture of hospitality, serving a meat-based dish is a widespread tradition and is regarded as a symbol of the value placed on the guest. Furthermore, Eid al-Adha reinforces meat consumption as a religious and cultural practice. All these factors demonstrate that meat in Turkey is not merely viewed as a nutrient, but also as a social and cultural symbol. For these reasons, it is believed that artificial meat may not be widely accepted in Turkey.

It is anticipated that artificial meat will be adapted only to a limited extent in the context of Turkey. This expectation is rooted in a combination of socio-cultural and religious factors. In particular, the symbolic meanings embedded in religious rituals such as Sacrifice (Kurban) and Votive Offering (Adak) are thought to play a decisive role in shaping public attitudes toward artificial meat. According to the beliefs of many in Turkish society, during the Eid al-Adha (Kurban Bayramı), an animal is slaughtered and a portion of the meat is distributed to those in need. This ritual reinforces the values of social solidarity and sharing. An Adak (votive offering) is made in fulfillment of a personal wish; once the wish is granted, an animal is sacrificed and its meat is distributed, thereby completing the vow. In both cases, the presence of a live animal and the act of slaughter hold cultural and religious value that artificial meat cannot reproduce (31–34). In this context, it may be argued that artificial meat does not possess the value attributed to traditional meat.

Moreover, according to Islamic belief, the meat consumed must be halal. Halal meat must come from a permissible animal, and the Basmala (the invocation In the name of God, the Most Gracious, the Most Merciful) must be recited during slaughter (32, 35, 36). Uncertainties surrounding the Halal nature of artificial meat are believed to raise questions about its religious acceptability and influence consumer preferences. In addition, the awareness of environmental sustainability is still in the early stages of development in Turkey (37), and the limited public knowledge regarding the positive ecological impacts of artificial meat is thought to influence consumer decision-making.

## 5 Conclusion and evaluation

The results obtained from the present study demonstrated that the majority of the participants were not knowledgeable enough about artificial meat. The analysis of the participants’ artificial meat consumption preferences demonstrated that a significant portion of the participants was not in favor of consuming artificial meat, and nearly half of them had a negative view of artificial meat. On the other hand, a small number of the participants might have a positive approach to artificial meat due to their perspectives of environmental sustainability and reduction of animal use. These results are not consistent with those of another study (38) in which consumer opinions were investigated. In that study, more than half of German consumers considered new food products promising and acceptable, and the majority of them were willing to try such products. Among the main factors influencing these attitudes of German consumers are probably ethical concerns, a sense of curiosity and environmental friendliness. In line with these results, it was concluded that the individuals participating in the present study were not knowledgeable of the production purposes of artificial meat and its necessity in terms of environmental sustainability. However, some of the participants, although few in number, were aware of the benefits of artificial meat in terms of environmental sustainability.

In another study conducted on artificial meat consumption preferences in African countries (39), the proportion of individuals willing to try artificial meat was also low. Only a small percentage of respondents considered artificial meat as a viable alternative in the future. Furthermore, of the participants in that study, those from wealthy and educated countries were more willing to try artificial meat. According to the results obtained in that study, ethical concerns were cited as the primary reason for willingness to try artificial meat, while safety concerns were cited as the primary reason for reluctance. The results of that study are consistent with those of the present study, which demonstrates the importance of raising awareness about artificial meat.

Studies on artificial meat consumption were also conducted in Brazil and China. According to the results of these studies, 40% of Brazilian participants and 47.2% of Chinese participants did not want to consume artificial meat regularly (40, 41). In a cross-country comparison, African and Chinese consumers had less emotional resistance to consuming artificial meat than did French and Brazilian consumers, which can be attributed to the more open-mindedness and the greater diversity of dietary cultures of African and Chinese consumers, including insect protein alternatives in Africa and plant protein alternatives in China (39). The cautious or unwilling stance toward artificial meat consumption in Turkish society is largely attributed to cultural and religious values.

According to the other results of the present study, the majority of the participants who adopted the Minimalist Lifestyle also adopted sustainable nutrition. Thus, it can be said that the characteristics of the minimalist lifestyle overlap the characteristics of the sustainable lifestyle. The scores obtained from the scales according to the preferring to consume artificial meat variable demonstrated that not all the participants associated this with environmental sustainability. While some of the participants thought that artificial meat was beneficial in terms of environmental sustainability, some other participants thought that artificial meat would be an economical and alternative protein source to feed the increasing world population today or in the near future. On the other hand, most of the participants were concerned that artificial meat could be harmful to human health. Considering the data obtained in the present study and ongoing research on artificial meat, it is predicted that artificial meat will gradually start to be used in the world due to the increasing world population. It is predicted that, in the forthcoming years, the issue of artificial meat will be addressed not only with its social, economic, health, positive and negative aspects but also with its environmental sustainability, and that ethical (bioethical) problems and dilemmas will arise. It is thought that with the increasing world population and decreasing world resources, which will shape consumption and nutrition preferences and will become a serious necessity in the coming years, if current consumption trends continue in the same way, it is predicted that sufficient resources will not be provided not only for future generations but also even for today’s generations. The present and similar studies are expected to contribute to both public opinion on the subject, and to raise awareness among both research participants and readers. Furthermore, the present and similar studies will help understand the attitudes that shape society’s consumer identity and, consequently, contribute to the development of policies and strategies focused on environmental sustainability.

In addition, considering the educational characteristics of the sample group included in the present study, most of whom had university education or master’s degree, it would be more appropriate to conduct studies with individuals with different educational characteristics to obtain in-depth data on the subject. It is suggested that future studies use probability-based or mixed recruitment strategies. Also conducting longitudinal studies is also believed to be beneficial in understanding how people’s views have changed over time. It is expected that the topic of artificial meat will continue to be relevant, and therefore, research in this area will continue, which will make it possible to determine the views of the public on the subject over time.

## Data Availability

The original contributions presented in the study are included in the article/supplementary material, further inquiries can be directed to the corresponding author.
